# Time consumption for non-conveyed patients within emergency medical services (EMS): A one-year prospective descriptive and comparative study in a region of Sweden

**DOI:** 10.1371/journal.pone.0251686

**Published:** 2021-05-13

**Authors:** Frida Malm, Annika Elfström, Emma Ohlsson-Nevo, Erik Höglund

**Affiliations:** 1 Department of Emergency Care, Örebro University Hospital, Örebro, Region Örebro County, Sweden; 2 University Health Care Research Center, Faculty of Medicine and Health, Örebro University, Örebro, Sweden; Technion - Israel Institute of Technology, ISRAEL

## Abstract

**Introduction:**

Over time, ambulance assignments have increased in number both nationally and internationally, and a substantial proportion of patients encountered by emergency medical services are assessed as not being in need of services. Non-conveying patients has become a way for emergency medical services clinicians to meet this increasing workload. It has been shown that ambulances can be made available sooner if patients are non-conveyed, but there is no previous research describing the factors that influence the non-conveyance time.

**Study objective:**

To describe ambulance time consumption and the factors that influence time consumption when patients are non-conveyed.

**Methods:**

A prospective observational review of 2615 non-conveyed patients’ ambulance and hospital medical records was conducted using a consecutive sample. Data were analysed with the Kruskal-Wallis test, Mann-Whitney U test and Spearman’s rank correlation (rho) for linear correlations.

**Results:**

The mean NC time for all ambulance assignments during the study period was 26 minutes, with a median of 25 minutes. The shortest NC time was 4 minutes, and the longest NC time was 73 minutes. NC times were significantly faster during the day than at night.

**Conclusions:**

This study provides new knowledge about time consumption when patients are non-conveyed. Although there are time differences when patients are non-conveyed, the differences observed in this study are small and not of clinical value. Ambulances will most often become available sooner if patients are non-conveyed. Although patients might be eligible for non-conveyance, policy-makers might have to decide when it is appropriate to non-convey patients from time, resource, patient safety and patient-centred care perspectives.

## Introduction

Ambulance assignments have increased over time both nationally and internationally [1, 2], and a substantial proportion (16–51%) of patients encountered by emergency medical services (EMS) are assessed by EMS clinicians as not being in need of ambulance care [2–5]. The reasons for this increase are still not well known, but some propose that an ageing population and an increasing proportion of elderly and multimorbid patients are part of the increasing demand for EMS [1, 6]. This growing and changing demand puts pressure on both ambulance and acute care systems to have the resources to care for the critically ill and injured. In an attempt to handle this changing demand, EMS are non-conveying patients to levels of care other than ambulance care and emergency department (ED) attendance [7, 8]. The term non-conveyance (NC) means that patients–with the help of guidelines and triage systems–are non-conveyed by the EMS to other levels of care other than ambulance care to the ED. More suitable healthcare options for non-conveyed patients can be self-care at home, primary health care, other healthcare facilities or taking alternate or own transportation to the ED. Guidelines differ across ambulance organizations both nationally and internationally because there is no consensus regarding how and whom to non-convey. Comparing non-conveyance assignment data and outcomes between different non-conveyance systems is difficult due to the differences in health care structure, competence, guidelines and patient-level factors. Some organizations do not allow patients to be non-conveyed, while others have developed decision support tools containing both guidelines and triage systems for NC [5, 7–9]. Several triage systems exist, such as the Manchester triage scale (MTS), the Emergency Severity Index (ESI), Adaptive process triage (ADAPT), the Taiwan Prehospital Triage System (TPTS) and the Rapid emergency triage and treatment system (RETTS) [10–14]. RETTS is the triage system most commonly used by Swedish EMS [15]. RETTS combines Emergency Signs and Symptoms (ESS) codes with vital signs to generate the final triage outcome. The assessment contains measurements of vital signs, including blood pressure, heart rate, respiratory rate, saturation, temperature and level of consciousness. The patient’s main complaint, description of illness and signs of symptoms can be sorted under a specific ESS code and triage level. Patients are triaged in accordance with RETTS as red, orange, yellow, green or blue, and the colour scheme determines within what time frame the patient should meet a physician. Red indicates a need for instant physician contact, and orange triaged patients can wait 20 minutes after hand-over at the ED. Both yellow and green mean that patients are not in immediate need of acute care and can wait. The final triage is decided depending on which factor produces the highest triage colour, either vital signs or ESS code [14, 15]. The RETTS was not designed for NC decisions and it is not validated for prehospital use [14].

Today, ambulances in Sweden are staffed with registered nurses, who often have a master’s degree in emergency medical care. In this paper, these nurses are referred to as EMS clinicians. Their increased level of competence enables them not only to perform qualified assessments and provide advanced on-site treatment but also to make decisions to non-convey patients [16]. NC decisions might affect the ambulance job cycle time [17, 18].

Few studies have described what affects the duration of ambulance assignments when patients are non-conveyed. Internationally, prolonged ambulance turnaround time after the ambulance has arrived at the ED has been described by EMS as a problem because it delays ambulance resources from becoming available for new assignments [5, 17, 19].

Turnaround times have been described at 35–53 minutes, but vary between systems. Variations in turnaround times have been described due to ED crowding and the interaction between the ambulance and ED personnel [20, 21]. There also seem to be shorter job cycle times when patients are non-conveyed [18], partly explained by the handover time at the ED.

This study explores a field of NC where there is little to no previous research. This description of what affects NC time consumption could help ambulance organizations and policy-makers when deciding how to design NC guidelines so that ambulance resources are available for the most critically ill and injured.

The aim of this study was to describe NC time consumption and what affects time consumption when patients are non-conveyed.

## Materials and methods

The regional guidelines for non-conveyance were implemented in 2015 and designed to be restrictive, finding as many patients as possible that risked deterioration. The guidelines gave registered and specialist nurses the mandate to independently non-convey patients.

According to the non-conveyance guidelines, all patients seen but not transported by the regional EMS are considered non-conveyed. Unless the patient refuses care, refuses transport, or refuses to be examined to such an extent that the EMS clinician can assess the level of care. The regional guidelines state that all patients, both children (<18 years) and adults (≥18 years), can be non-conveyed to different levels of care, the lowest being self-care, followed by primary health care and ED via personal or public transport. After a structured patient interview, measurements of all vital signs (blood pressure, heart rate, respiratory rate, oxygen saturation, temperature and level of consciousness) and fulfilment of the region specific guideline criteria for non-conveyance, adult patients can be eligible for NC if triaged to have normal (green) vital signs and an ESS code of green or yellow according to RETTS. For children to be considered for non-conveyance, both the vital signs and ESS code must be triaged as green.

The region specific guidelines exclude patients from non-conveyance if they require drug administration (for instance, intra-venous pain medicine), supervision, or monitoring during transport (for instance, ECG or vital signs). All patients must be able to communicate and understand the decision and information provided (and not be under the influence of alcohol). For children, a legal guardian must be present.

**Fig 1:**
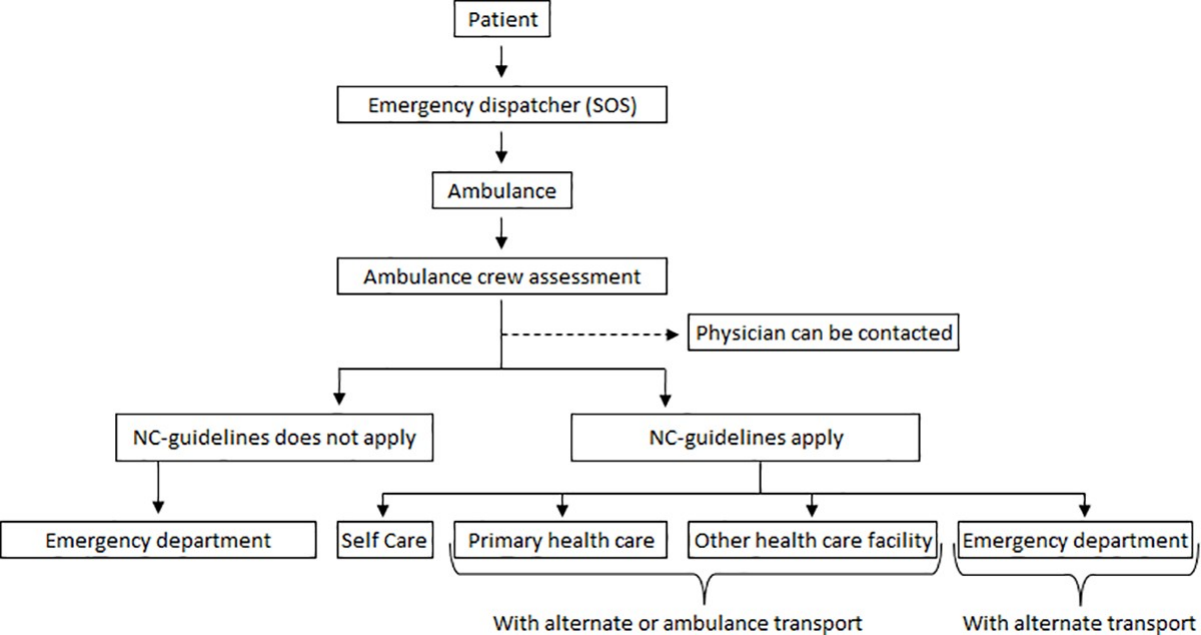
Flow chart for non-conveyed patients in the study [22].

To non-convey patients to a facility with the right competence and equipment, the clinicians have a list of conditions appropriate for primary healthcare services. A physician could be contacted for support. A non-conveyed patient could be referred to self-care at home, to primary healthcare, or to the ED with patient transport or with their own transportation. Patients could also, if appropriate, be assisted in booking transport or acquiring a primary healthcare appointment (Fig 1).

All non-conveyed patients received information and a document explaining which level of care was medically indicated. The document also contained information about where the patient could turn for healthcare advice or what to do if the condition worsened. The patient could also receive help contacting other healthcare services.

### Design

This was an observational study with consecutive and prospective inclusion of patients’ medical records after non-conveyance by the EMS. The report follows the Strengthening the Reporting of Observational Studies in Epidemiology (STROBE) guidelines [23].

### Setting

The studied region in Sweden has a population of 295 000 spread over 8 504 km^2^. There are three EDs in the region, with 90 000 visits per year. The three hospitals have their own ambulance departments. Together, approximately 30 000 assignments per year are divided between one larger and two minor stations. Of these 30 000, approximately 12% were non-conveyed during the study period. The majority of all assignments are for patients situated within 10 km of the receiving healthcare facility, and the approximate longest ambulance transport distance in the studied region is 80 km. The overall job cycle time for primary ambulance assignments in the studied region was 57 minutes, with a turnover time of 20 minutes and an ambulance on-site time, when a patient is assessed before transport, of 18 minutes.

### Data collection

All ambulance records of non-conveyed patients between February 2016 and February 2017 were collected for this study. Data extracted from the records were patient age, sex, ESS coding, time of arrival and departure from the patient’s side, and time of day. Data from all EMS clinicians working in the region during the study period regarding gender, age, work experience and education level were collected and recorded.

**Fig 2:**
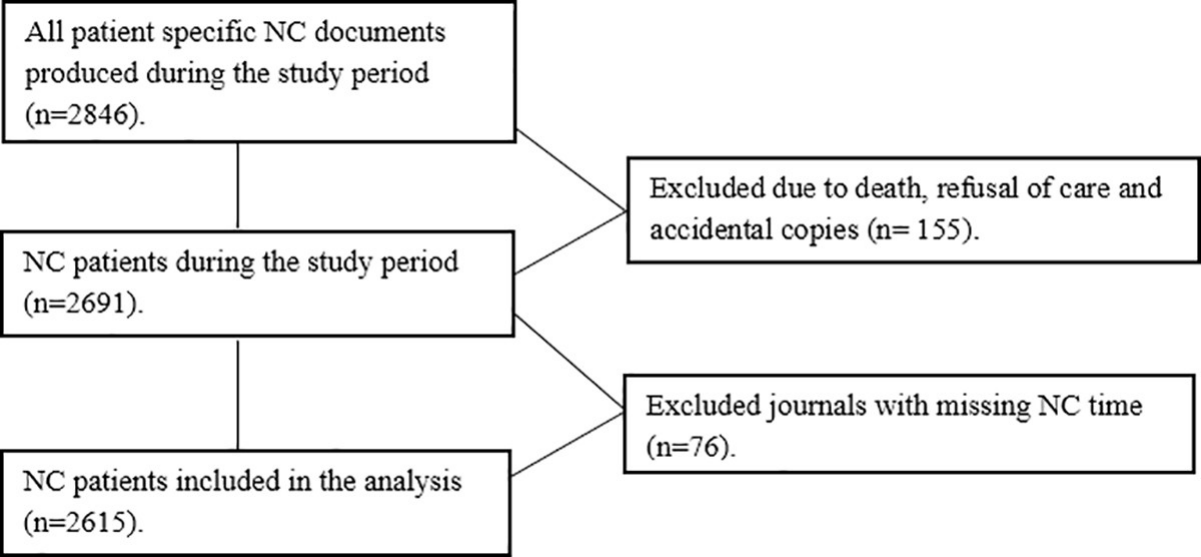
Included and excluded patient records.

The inclusion criteria were all patients non-conveyed during the study period where an NC record could be retrieved. Records were excluded for patients who died on site or refused care; accidental copies and records with missing NC times were also excluded (Fig 2).

The data gathering procedure was verified both during inclusion and afterwards using statistical methods. For instance, variables had fixed options to minimize the risk of ambiguous values. Each variable was also checked for values that were outside the range for that specific value. Before the analysis started, the accuracy of the data transfer from paper records to data files was evaluated. In total, 30% of all transferred data were manually checked for accuracy. The records were divided between the first author and two research administrators. Randomly selected records were checked against the database for accuracy. If any of the variables were incorrect, that variable was corrected in the database. The medical record before and after the record containing an incorrect variable was also checked for accuracy. After manual checks and corrections, transfer incorrectness was <0.25%.

### Data analysis

Demographic data are presented with frequencies and proportions for categorical variables and means, medians, interquartile range and standard deviations (SD) for continuous variables. The Kruskal-Wallis test was used for comparisons of age, gender, experience and education level, minor and major ambulance stations, time of day, ESS and number of NC patients per EMS clinician. For groups >2 where the Kruskal-Wallis test was significant, the Mann-Whitney U test was used. Spearman’s rank correlation (rho) was used for linear correlations of age, time of day and number of NC patients per clinician. Winsorizing was used to address outliers [24]. In total, 17 outliers were adjusted to 2.5 x SD. ESS codes containing fewer than 10 patients were analysed together as a single group presented as “Others”. Data were analysed using IBM SPSS Statistics version 22.

**Fig 3:**
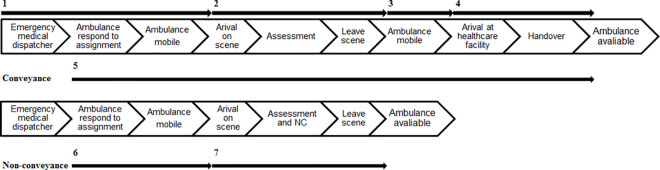
Timeline for ambulance assignments. Response time (time between when the call connected until an ambulance arrived on the scene).Time on scene (time from arrival on the scene, through the assessment, until the ambulance left the scene).Time to the healthcare facility (transportation time with a patient to a healthcare facility).Turnaround time (time at the healthcare facility, including making the resources available for new assignments).Job cycle time (time from when the ambulance crew received the assignment until the ambulance was available again).Same as 1. Response time (time between when the call connected until an ambulance arrived on the scene).NC time (including getting to, assessing, NC and leaving without the patient).6+7. Non-conveyance job cycle time (time from when the ambulance crew received the assignment until the ambulance was available again).

A P-value threshold <0.05 was used to measure statistical significance. The time it takes to non-convey a patient (NC time) was calculated as the difference between ambulance arrival on scene until the ambulance left the scene (Fig 3).

### Measured variables

The measured variables included the NC time (described for both the age and gender of the patients and EMS clinicians), time of day, clinician education level and workplace, number of NC patients per EMS clinician and ESS codes (Table 1).

**Table 1:** Measured variables.

Time of Day	Patient	EMS clinicians
	Gender	Gender
	Age	Age
	ESS code	Professional experience and education level
		Major or minor hospital and ambulance station
		Number of NC per EMS clinician

### Ethics statement

This study follows the approval of the ethical regional review board in Uppsala, Sweden (Dnr: 2015/465) and the ethical principles of the Helsinki declaration [25]. The medical records were available only for the research group and the research administrators at the regional research centre, who manually entered the data into the study-specific database. According to the laws and regulations for research data, all medical records were then securely stored for ten years. The study-specific database was fully anonymized after completion, and the results were reported only on a group level. The research committee waived the requirement for informed consent.

## Results

### Patient demographics

A total of 2615 NC patients (Fig 1) were included in the study. Gender was distributed as follows: 1302 (49.8%) males, 1285 (49.1%) females and 28 (1.1%) unknown gender. Patients of all ages (from newborns to 99 years old) were non-conveyed, with a mean age of approximately 50 years (Table 2).

**Table 2:** Patient demographic data and NC times.

		Number of patients (%)	Time in minutes (mean/median)	Time in minutes q1-q3 (SD)
**Total**		2615 (100)		
	Range		4–73	
	(Mean/median)		26/25	
	(q1-q3)		18–32	
**Gender**	Male	1302 (49.8)	(25/24)	17–31 (11)
	Female	1285 (49.1)	(26/25)	19–32 (11)
	Missing	28 (1.1)	(28/25)	15–33 (13)
**Age (years)**	Mean	49.6		
	Median	52.0		
	Range	0–99		
	q1-q3	25–73		
**Age**	0–10	263 (10.1)	(25/24)	18–30 (10)
**category**	11–17	110 (4.2)	(23/21)	16–28 (11)
	18–30	460 (17.6)	(23/21)	15–30 (10)
	31–45	316 (12.1)	(24/23)	18–30 (10)
	46–64	474 (18.1)	(25/25)	18–32 (11)
	65–80	561 (21.5)	(27/25)	20–33 (12)
	>80	431 (16.5)	(28/27)	20–35 (12)
**Time of day**	00:00–05:59	444 (17.0)	(28/25)	20–35 (12)
	06:00–11:59	555 (21.2)	(25/24)	17–32 (11)
	12:00–17:59	745 (28.5)	(25/23)	17–30 (11)
	18:00–23:59	871 (33.3)	(26/25)	18–32 (11)

### NC time

The mean NC time for all ambulance assignments during the study period was 26 minutes, with a median of 25 minutes q1-q3 (18–32, range 4–73). Male gender and older age were associated with longer NC times (Table 2). The shortest NC time was four minutes, and the longest NC time was 73 minutes. Daytime was significantly faster than nighttime, with means of 25 and 28 minutes, respectively.

### EMS clinicians

In total, 149 EMS clinicians, 69% male and 31% female, chose to non-convey patients during the study period. For all clinicians, age ranged between 24–65 years, work experience from 0–39 years, and 56% had a Master’s degree in prehospital care. The EMS clinicians chose to non-convey between one and 58 patients each, with an average of 17 patients per clinician. Almost 40% of all NC decisions were made by 25 EMS clinicians. All of these 25 clinicians non-conveyed more than 30 patients. There were more patients non-conveyed in the major city (n = 1791), than in both the minor cities together (n = 824). The EMS clinicians were evenly distributed between the major city ambulance department (76, 51%; 73% male and 27% female) and the two minor city ambulance departments (73, 49%; 70% male and 30% female). EMS clinician specialist education in the major city was 74% and in the minor cities, it was 38% (Table 3).

**Table 3:** Demographic data and NC times of the EMS clinicians that had non-conveyed patients during the study period.

		n (%) (mean/median)	Range	Time in minutes (mean/median)	Time in minutes q1-q3 (SD)
**EMS clinicians**		149 (100)			
**Gender**	Male	103 (69.1)		(25/24)	18–32 (11)
	Female	46 (30.9)		(26/25)	19–32 (11)
**Age (years)**		(43.1/42.0)	24–65		
	24–29	14 (9.4)		(25/23)	17–30 (11)
	30–39	50 (33.6)		(25/24)	18–30 (10)
	40–49	40 (26.8)		(25/25)	18–31 (11)
	50–59	33 (22.1)		(27/25)	20–35 (12)
	60+	12 (8.1)		(26/24)	18–33 (11)
**EMS clinicians per ambulance station**	Major city	76 (51.0)		(24/23)	17–30 (10)
	Minor city	73 (49.0)		(28/26)	20–35 (12)
**Education**	EMS clinician	149 (100)		(26/25)	19–32 (12)
	EMS clinician specialist	84 (56.4)		(25/24)	18–31 (11)
**Numbers of referrals per EMS clinician**		(18.0/16.0)	1–58		
	1–30	124 (83.2)		(27/25)	19–34 (11)
	>30	25 (16.8)		(24/22)	16–30 (10)

No significant difference in NC time was demonstrated between genders and among education levels. A longer NC time was associated with fewer NC decisions and a clinician age of 50–59.

### Emergency signs and symptoms (ESS)

According to ESS coding, the main complaints that showed the longest NC times were epistaxis and hypoglycaemia for adult patients. Traffic accidents and fainting/syncope were the ESS codes that took the least amount of time on site. For children, fever and missing ESS had the longest NC times, and hip, leg or foot injury, and swollen groin, abdominal pain or groin pain were the two fastest NC symptom groups (Table 4).

**Table 4:** NC time according to the main complaint for adults and children.

Main complaint	Number of patients (%)	Time in minutes (mean/median)	Time in minutes q1-q3 (SD)
Adults			
80—Traffic accidents*	53 (2.0)	*(18/17)	13–22 (06)
20—Fainting, syncope	78 (3.0)	*(21/17)	14–29 (10)
35—Burn, inhalation or chemical damage	14 (0.5)	(21/18)	15–33 (12)
34—Hip, leg or foot injury	71 (2.7)	*(21/20)	15–25 (07)
41—Bite or toxic effect of animals	12 (0.5)	(21/23)	13–27 (07)
33—Hand, arm or shoulder injury	50 (1.9)	*(22/18)	14–27 (14)
19—Headache	48 (1.8)	(23/21)	13–30 (11)
40—Poisoning by alcohol or others	86 (3.4)	*(23/21)	15–30 (10)
9—Convulsions	45 (1.7)	(23/21)	17–27 (08)
16—Urine problem	25 (1.0)	(24/23)	16–29 (09)
30—Head or neck injury	77 (2.9)	(24/25)	15–30 (10)
47—Fever or infection	65 (2.5)	(24/24)	17–30 (08)
31—Back or pelvis injury	35 (1.3)	(24/25)	17–30 (10)
53—Others in ESS 53 *	120 (4.6)	(25/21)	15–33 (13)
6—Abdominal pain, nausea or diarrhoea	199 (7.7)	(25/23)	19–30 (09)
83—Inclined to fall *	47 (1.8)	(25/24)	18–30 (09)
12—Stroke or neurological loss	29 (1.1)	(25/24)	18–31 (11)
43—Allergic reactions	39 (1.5)	(25/25)	16–30 (10)
11—Dizziness	115 (4.4)	(25/25)	18–33 (10)
99—Others **	50 (1.9)	(26/25)	17–32 (10)
15—Pain or swelling in extremity	48 (1.8)	(26/25)	19–29 (09)
5—Chest pain	126 (4.8)	(26/25)	20–30 (09)
21—Women’s genital disorders or pregnancy	22 (0.8)	(27/25)	18–35 (13)
4—Dyspnoea	159 (6.1)	(27/25)	19–34 (12)
0—Missing ESS	80 (3.2)	(27/25)	20–32 (11)
49—Hyperglycaemia or others; diabetes	14 (0.5)	(28/25)	18–4) (11)
81—Malaise *	184 (7.0)	*(28/26)	20–35 (12)
52—Psychiatric problems *	71 (2.7)	*(29/26)	20–40 (12)
2—High or low blood pressure	12 (0.5)	(30/24)	20–41 (13)
1—Abnormal heart rhythm	45 (1.7)	*(31/28)	20–37 (14)
14—Back pain/Thoracic pain	64 (2.4)	*(32/30)	23–40 (13)
50—Hypoglycaemia	108 (4.1)	*(33/30)	25–42 (14)
3—Epistaxis	51 (2.0)	*(34/32)	24–40 (12)
**Total**	**2242 (85.8)**		
**Children**			
134 Hip, leg or foot injury	20 (0.8)	*(18/18)	13–24 (06)
106 Abdominal or groin pain, swollen groin	19 (0.7)	*(20/20)	19–24 (04)
130 Head or neck injury	32 (1.2)	(21/20)	16–26 (08)
146 Nose problems or foreign body in nose, throat or gastrointestinal tract.	13 (0.5)	(21/23)	14–26 (10)
153 Other malaise	31 (1.2)	(23/20)	17–30 (10)
999 Others **	79 (3.0)	*(23/22)	15–30 (10)
143 Allergic reactions	20 (0.8)	(25/22)	19–33 (10)
104 Dyspnoea	56 (2.1)	(26/24)	17–34 (12)
144 Sore throat, coughing and colds	17 (0.7)	(26/25)	19–31 (08)
147 Local infection	11 (0.4)	(27/25)	19–32 (12)
109 Convulsions	26 (1.0)	(27/25)	19–34 (10)
100 Missing	14 (0.5)	(28/26)	18–40 (14)
154 Fever	35 (1.3)	*(30/30)	22–35 (10)
**Total**	**373 (14.2)**		

* Significantly different from the population mean, p<0.05.

## Discussion

The primary objective of this study was to investigate the time elapsed when patients are non-conveyed by EMS clinicians.

The overall results show significant differences in various time aspects of NC. These time differences are relatively small and therefore probably clinically irrelevant. Previous studies showed significant time differences between non-conveyed and conveyed patients [19], with mean ED turnaround times over 35 minutes [21]. Ambulance turnaround has been described as a problem that hinders the availability of ambulance resources [17, 19]. The Department of Health in the UK has set time targets for both the time at the patient (15–20 minutes) and ED turnaround time (<15 minutes). Furthermore, the Department of Health in the UK has noted that activities that do not add value or that lead to delays must be avoided and that processes must be simplified and move faster [26]. It is possible that the overall job cycle time could be shortened if eligible patients are non-conveyed because ambulance resources will most often become available immediately after the patient is non-conveyed.

For adult patients, epistaxis and hypoglycaemia rendered the longest NC times. Both epistaxis and diabetic patients often require care interventions [27, 28]. The EMS clinician might need to confirm that the patient’s mental readiness has been restored, rule out other acute underlying causes for the symptom presentation and understand the patients and their surrounding supportive capabilities. Diabetic patients with low blood sugar, for instance, could take longer to non-convey because they often need intravenous glucose, and both intravenous access and glucose administration take time. Furthermore, the patient interview could be delayed because it takes time for patients with low levels of blood sugar to regain full mental status [27, 29], which is a requirement for NC.

For younger patients under the age of 10, fever was the ESS code associated with the longest NC time. Children presenting with symptoms of illness pose a challenge when making NC decisions because they often cannot communicate adequately for themselves, parents need to be relieved of their concerns [30, 31], and healthcare personnel consider children to be a greater responsibility than adults [32, 33].

The results show that higher NC frequencies for EMS clinicians were significantly associated with male gender. The difference could not be explained by education, experience or age. It has been shown that male nurses are predominately exposed to disciplinary actions and license suspension [34], which could be interpreted as a sign of risk-taking.

The number of patients non-conveyed by each EMS clinician ranged between one and 58 patients per year.

Some EMS clinicians who worked in the region during the study period did not non-convey any patients. It was not possible to extract data on how much each clinician worked or how many patients eligible for NC were encountered by each clinician during the study period. Not knowing how much each clinician worked and how many patients they met who were eligible for non-conveyance might explain some of the differences observed in the number of patients non-conveyed by each clinician.

Not feeling support from the organization, reducing one’s own liability and trying to become available faster for patients with potentially more acute care needs have all been proposed as reasons for not adopting the guidelines [22], and instead, the EMS clinicians choose to transport the patients to the ED. The regional guidelines state that it is not imperative to adopt the guidelines, even if patients are eligible for NC. This might affect the equality of care and ambulance availability, and thus might result in patients attending the ED without needing its services.

EMS clinicians adopting the guidelines more frequently non-conveyed patients significantly faster. These clinicians might become familiar with the NC guidelines or get better at assessing patients suitable for NC and therefore could reduce the time needed. However, it is also possible that they were faster because they did not fulfil all NC requirements. There are no guidelines for how long a prehospital assessment (including triage with RETTS) should take. It is reasonable to question whether NC times under 10 minutes are consistent with a safe patient assessment. Likewise, an unreasonably long NC time could be questioned because an acute care resource is occupied for a long period. Patient safety and the patient’s own experience of the NC must be considered. Ambulance organizations and policy-makers might have to decide whether time consumption and ambulance availability should be a factor when guidelines are created and NC decisions are made.

Although it appears that this study collected all produced NC records during the research period, it is likely that more patients could have been non-conveyed because EMS clinicians might sometimes choose to disregard the NC guidelines. This calls for further studies and educational efforts to ensure that EMS clinicians will use the guidelines as intended.

A non-parametric test was used to analyse the data because some of the subgroups did not meet the criteria for parametric tests. All analyses were, when suitable, compared with parametric tests, and they corresponded with the non-parametric tests.

It can be argued that handwritten record data in some cases could be misinterpreted. However, even if this could have happened, we emphasize that it would not change the statistical calculations or results because the margin for error is negligible. Instead, time estimates could impose error because data are handwritten. This has been shown in the data because whole numbers are overrepresented in the dataset. However, according to statistical analysis, this should also not pose a risk because the error seems–and should logically be–as much over- as underestimated. The alternative could have been to use only the manually entered digital time that is produced when the ambulance crew considers themselves to be available for a new assignment and is returning to the station, although these time marks hold the same margin for error and ambiguous data. However, we argue that we have used the best available data because no one other than the ambulance crew can decide when the ambulance resource is available for new assignments. The relatively large data set also makes it reasonable to assume that individual time estimations would not affect the results as a whole.

However, because there was no difference in the NC time when ambulance specialist education level was taken into account, it is possible that specialist education, in general, does not reduce time consumption.

The mean ambulance job cycle time and patient assessment time were retrieved from the same database and for the same year as the initial data collection for the study. The mean time used contains only primary ambulance missions where a patient was assessed on site, transported to the ED, or non-conveyed.

Some ESS codes had to be grouped together because they contained too few NC decisions. It is possible that these codes are used for more severe conditions and for patients with comorbidities that make the NC time longer.

## Conclusions

Although there are significant time differences between patient and EMS clinician characteristics, we believe that the differences in time consumption when patients are non-conveyed are negligible. The ambulance resource could become available sooner if patients are non-conveyed. Ambulance availability cannot be seen as the only parameter of value because it can be argued that patient satisfaction, compliance, costs and resource availability are also important components.

It is important to understand that faster NC times cannot be a goal in itself because when taken out of context, it conveys no information about patient safety, satisfaction and resource efficiency.
